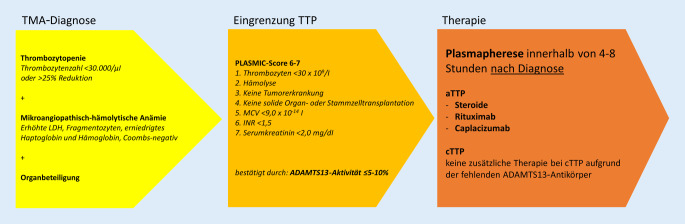# Thrombotisch-thrombozytopenische Purpura – eine differenzialdiagnostische Herausforderung im Notfall

**DOI:** 10.1007/s00063-022-00982-w

**Published:** 2023-01-04

**Authors:** Fedai Özcan, Martin Köhrmann, Sirak Petros, Andreas Goette, Peter Bramlage, Martin Bommer, Jörg Christian Brokmann

**Affiliations:** 1 Klinik für Nephrologie, Klinikum Dortmund, Universität Witten-Herdecke, Beurhausstr. 40, 44137 Dortmund, Deutschland; 2 Klinik für Neurologie, Universitätsklinikum Essen, Essen, Deutschland; 3 Interdisziplinäre Internistische Intensivmedizin, Universitätsklinik Leipzig, Leipzig, Deutschland; 4 Department of Cardiology and Intensive Care Medicine, St. Vincenz-Hospital, Paderborn, Deutschland; 5 Institut für Pharmakologie und Präventive Medizin, Cloppenburg, Deutschland; 6 Klinik für Hämatologie, Onkologie, Palliativmedizin, und Infektionskrankheiten, Alb-Fils-Kliniken, Göppingen, Deutschland; 7 Zentrale Notaufnahme, Uniklinik RWTH Aachen, Aachen, Deutschland

**Keywords:** Thrombotisch-thrombozytopenische Purpura (TTP), LDH Erhöhung, Organbeteiligung, ADAMTS13-Aktivität, Plasmapherese, Thrombotic thrombocytopenic purpura (TTP), LDH elevation, Organ involvement, ADAMTS13-activity, Plasmapheresis

## Abstract

Das Vorliegen einer thrombotisch-thrombozytopenischen Purpura (TTP) als Kombination aus Thrombozytopenie, LDH-Erhöhung und Anämie in Kombination mit einer Organbeteiligung ist eine seltene, aber lebensbedrohliche Erkrankung, die unbehandelt mit einer extrem hohen Letalität in der Akutphase einhergeht. Wir stellen in dem vorliegenden Beitrag den Fall einer 49-jährigen Patientin vor, die mit unklaren abdominellen Beschwerden und subfebrilen Temperaturen stationär aufgenommen wurde, und leiten daraus Empfehlungen für die Notfallsituation ab. Ein erhöhtes Bewusstsein für die Erkrankung und die zielgerichtete weiterführende Diagnostik mit Bestimmung des PLASMIC-Scores bzw. der ADAMTS13-Aktivität führt ggf. direkt zur TTP, deren verzögerte Diagnostik regelhaft zu Sekundärkomplikationen führen würde.

## Abstract (en)

Thrombotic thrombocytopenic purpura (TTP), which comprises thrombocytopenia, elevated lactate dehydrogenase levels, and anemia in the combination of organ involvement, is a rare but life-threatening condition associated with an extremely high lethality in the acute phase if left untreated. Using the example of a 49-year-old woman admitted to the hospital with unexplained abdominal symptoms and subfebrile temperatures, recommendations for the emergency situation are presented. Increased awareness of the disease and targeted further diagnostics with determination of the PLASMIC score or ADAMTS13 activity may lead directly to diagnosis of TTP; delayed diagnosis is usually associated with secondary complications.

## Zielsetzung

Diese Übersicht soll …

- die Assoziation der Laborkonstellation aus Thrombozytopenie, LDH-Erhöhung und Anämie mit der Diagnose einer thrombotischen Mikroangiopathie herausarbeiten;
- die Unterscheidung der thrombotisch-thrombozytopenischen Purpura (TTP) von den Differenzialdiagnosen atypisches hämolytisch-urämisches Syndrom (aHUS) und Shiga-Toxin-assoziertes HUS ermöglichen:
- die Bedeutung des PLASMIC-Scores als frühen Hinweis auf das Vorliegen einer TTP herausarbeiten und
- die Notwendigkeit einer frühzeitigen, gerichteten spezifischen Therapie betonen.

## Fallvorstellung, Diagnostik

Eine 49-jährige Patientin wurde initial mit unklaren abdominellen Beschwerden und subfebrilen Temperaturen stationär aufgenommen. In der Vorgeschichte war bei der Patientin ein Ovarial- bzw. Tubenkarzinom aus dem Oktober 2014 bekannt. Es wurde damals eine Hysterektomie, Adnexektomie sowie eine pelvine und paraaortale Entfernung der Lymphknoten durchgeführt. Eine Thrombozytopenie lag zum Zeitpunkt der Aufnahme nicht vor, die Nierenfunktion war regelgerecht, die Bestimmung der Laktadehydrogenase (LDH) im Serum wurde nicht angefordert.

Die Patientin entwickelte innerhalb von 5 Tagen einen Abfall der Thrombozyten auf 85.000 µ/l und an Tag 7 einen Hb-Abfall auf 7,5 g/dl (Normwert 12–16 g/dl), eine Thrombozytopenie mit 5000/l und ein LDH von 2987 U/l (Normwert 135–215 U/l). Das Kreatinin stieg auf 1,7 mg/dl. Zwei weitere Tage später (Tag 9) sank der Hb-Wert auf 4,9 g/l, das LDH lag bei 2062 U/l und es wurde ein Haptoglobinverbrauch (Haptoglobin 0,14 g/l bei einem Normwert von 0,30–2,00 g/l) gemessen. Im Blutausstrich waren reichlich Fragmentozyten (34 ‰) nachweisbar. Der Coombs-Test war negativ, die Thrombozytenzahl betrug 5000/µl (Normalbereich 150.000–80.000/µl). Sie war wach und orientiert, wirkte allerdings verlangsamt und berichtete später von Gedächtnisstörungen.

Es lag ein akutes Nierenversagen im Stadium I nach KDIGO vor. Das Serumkreatinin stieg passager auf maximal 1,7 mg/dl (Normwert 0,5–1,0 mg/dl). Das Troponin war mit 389 ng/l deutlich erhöht. Die Patientin wurde an Tag 9 in unsere Klinik zur weiteren Behandlung verlegt.

## Einordnung der Beobachtungen

**Figure 1:**
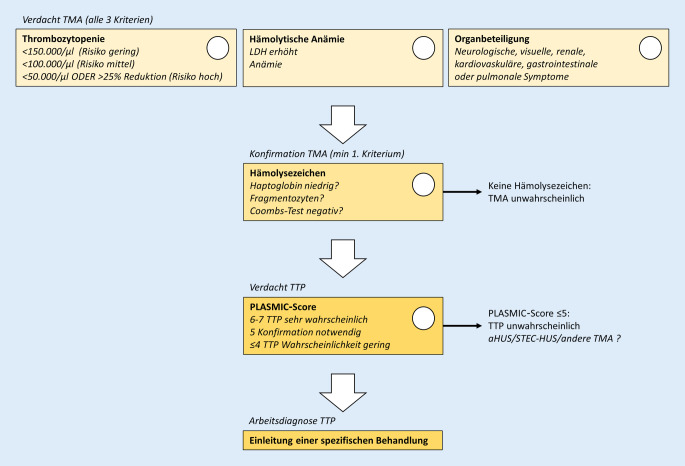

Aus der klinischen Bestandsaufnahme (Organbeteiligung; Tab. 1) sowie aus der Laborkonstellation Thrombozytopenie, LDH-Erhöhung (als Ausdruck einer evtl. Hämolyse und Organischämie) und Anämie ergibt sich bereits in der Notaufnahme die Verdachtsdiagnose einer thrombotischen Mikroangiopathie (TMA; Abb. 1). Differenzialdiagnostisch kommen bei abdominalen Schmerzen zusammen mit einer Troponinerhöhung auch der akute Myokardinfarkt, bei CRP-Erhöhung auch gastrointestinale Notfälle infrage.

**Table 1:** 

	Symptome
Neurologische Symptome	Quantitative/Qualitative Bewusstseinsstörungen und/oder epileptische Anfälle
Visuelle Symptome	Sehstörungen, akute Blindheit
Nierenschädigung	Erniedrigte GFR und/oder pathologischer Urinbefund, Hypertonie
Kardiovaskuläre Symptome	Myokardinfarkt, Angina pectoris
Gastrointestinale Symptome	Durchfall und/oder Übelkeit/Erbrechen, abdominale Schmerzen, Gastroenteritis
Pulmonale Symptome	Oxygenierungsstörung, Lungenblutung, Lungenödem

*GFR* glomeruläre Filtrationsrate

Die Thrombozytopenie ist Ausdruck eines gesteigerten Verbrauchs durch die intravasale Thrombenbildung. Die Dynamik der Erkrankung spiegelt sich vor allem auch in den rasch fallenden Thrombozytenzahlen. Diese wird im nächsten Schritt durch mindestens ein Hämolysezeichen (Fragmentozyten, erniedrigtes Haptoglobin) bei negativem Coombs-Test gesichert. Fehlen Hämolysezeichen, ist eine TMA unwahrscheinlich.

Unter dem Begriff der TMA wird eine heterogene Gruppe von Erkrankungen zusammengefasst, die typische pathologische Schädigungen des Endothels von Arteriolen und Kapillaren aufweisen und durch eine gesteigerte Inflammations- und Gerinnungskaskade zu einem (thrombotischen) Verschluss der kleinsten Gefäße führen. Es handelt sich um eine Systemerkrankung mit Organdysfunktion infolge der Mikroangiopathie, wobei die neurologischen und renalen Symptome im Vordergrund stehen. Die pathophysiologischen Mechanismen sind mittlerweile bekannt und charakterisiert. Es werden primäre und sekundäre Formen unterschieden.

Um an dieser Stelle, möglichst zielgerichtet, eine TTP auszuschließen, wird eine Aussage zur Aktivität von ADAMTS13 („a disintegrin and metalloproteinase with a thrombospondin type 1 motif, member 13“) benötigt. Eine erste Abschätzung der Aktivität ergibt sich aus dem *PLASMIC-Score* [1], der zur Prüfung der Verdachtsdiagnose TTP herangezogen wird (Tab. 2). Bei einem Score von 0–4 ist das Risiko für eine schwere ADAMTS13-Defizienz gering (0–4 %), bei einem Wert von 5 intermediär (5–25 %) und ab einem Score von 6 hoch (62–82 %). Liegt der Score unterhalb von 6, liegt im Normalfall keine TTP vor. Bei Werten von 5 oder mehr wird die ADAMTS13-Bestimmung und die Überweisung an/Beratung durch einen Spezialisten empfohlen [2]. Bei Werten unter 5 wird die ADAMTS13-Aktivität nur bestimmt, wenn keine Alternativerklärung vorliegt.

**Table 2:** 

	Nein	Ja
Thrombozytenzahl < 30.000/µl	0	*+1*
Hämolyse Retikulozyten > 2,5 % *oder* Haptoglobin nicht nachweisbar *oder* indirektes Bilirubin > 2,0 mg/dl (34,2 µmol/l)	0	*+1*
Aktive Tumorerkrankung Behandlung einer Tumorerkrankung innerhalb des letzten Jahrs	*+1*	0
Solide Organ- oder Stammzelltransplantation in der Anamnese	*+1*	0
Mittleres Erythrozytenvolumen (MCV) < 9,0 × 10^−14^ l (< 90 fl)	0	*+1*
International Normalized Ratio (INR) < 1,5	0	*+1*
Serumkreatinin < 2,0 mg/dl (176,8 μmol/l)	0	*+1*

Für die Laborwerte sollten die ersten verfügbaren Werte verwendet werden. Werte, die 72 h oder später nach Krankenhausaufnahme gewonnen werden, finden keine Verwendung

Ätiologisch liegt der TTP ein Abfall der ADAMTS13-Aktivität auf < 5–10 % [3] zugrunde. Folglich treten ultragroße, sehr adhäsive von-Willebrand-Faktor-Multimere in der Gefäßstrombahn auf, deren Nachweis die ersten wichtigen Hinweise für die pathophysiologischen Mechanismen der Erkrankung lieferte. In den allermeisten Fällen sind Antikörper gegen ADAMTS13 nachweisbar. Es handelt sich somit um eine erworbene, autoimmunvermittelte Erkrankung.

Bestätigt wird die Diagnose durch den Nachweis der erniedrigten ADAMTS13-Aktivität im Speziallabor. Allerdings liegen die Ergebnisse der ADAMTS13-Aktivitätsbestimmung aktuell im Mittel erst nach 3–5 Tagen vor, sodass die Verdachtsdiagnose einer TTP und damit die Einleitung einer Behandlung bereits aus der Konstellation einer Hämolyse mit Fragmentozyten und einer Thrombozytopenie sowie der möglichen Organbeteiligung abgeleitet werden muss.

Die TTP erfordert eine umgehende Behandlung innerhalb weniger Stunden. Es handelt sich um einen Notfall im engeren Sinne. Ähnlich wie bei der Sepsis hängt die Morbidität und Letalität wesentlich von einer raschen Diagnose und Einleitung einer adäquaten Behandlung ab. Thrombotische Organkomplikationen treten häufig in den ersten Stunden auf. Myokardiale Ischämien sind mit dem Risiko eines akuten Herztods, zerebrale Ischämien sind, neben der durch die Ischämie selbst bedingten Symptome, auch mit Psychosyndrom, epileptischen Anfällen und Koma assoziiert. Unbehandelt nehmen die anfangs flüchtigen neurologischen Symptome häufig einen progressiven Verlauf [4].

Im vorliegenden Fall waren sowohl Fragmentozyten als auch eine schwere Thrombozytopenie nachweisbar. Das erhöhte Troponin im Kontext der übrigen Laborparameter, der Kreatininanstieg und die temporären Gedächtnisstörungen waren als Hinweis auf eine Organbeteiligung mit einer TTP gut vereinbar. Zudem waren im vorliegenden Fall alle Laborkriterien des PLASMIC-Scores erfüllt, während eine aktive Tumorerkrankung bzw. eine vorhergehende Organ- oder Stammzelltransplantation ausgeschlossen wurde. Damit ergab sich ein PLASMIC-Score von 7, ein hohes Risiko für eine schwere ADAMTS13-Defizienz und die dringliche Indikation zur Behandlung.

## Diagnose

Die TTP präsentiert sich vor allem über eine hämolytische Anämie (100 %), Thrombozytopenie (100 %) und in der Regel (80 %) über neurologische Störungen (Kopfschmerzen, Verwirrtheit, Psychosyndrom, fokal-neurologische Defizite und epileptische Anfälle). Weiter zur Symptompentade gehören Fieber (10 %) und Niereninsuffizienz im Stadium 3 und höher (9 %). Wichtig ist dabei der Zeitverlauf: Eine Organbeteiligung manifestiert sich häufig früher als die schwere Hämolyse und ausgeprägte Thrombozytopenie.

Die TTP ist im Regelfall (95 %) erworben („acquired“ TTP oder aTTP), auch wenn sie mit 1,5–6 Fällen pro 1 Mio. Einwohner und Jahr absolut gesehen selten ist. Die angeborene Form (Upshaw-Schulman-Syndrom, „congenital“ TTP oder cTTP) wird dagegen für nur etwa 5 % aller TTP-Fälle verantwortlich gemacht. Wesentliches Unterscheidungskriterium ist der Nachweis von Anti-ADAMTS13-Antikörpern, die bei der aTTP pathognomonisch (aber nicht immer auch tatsächlich nachweisbar) sind, bei der cTTP jedoch grundsätzlich fehlen.

Die cTTP basiert auf einer genetisch bedingten Verminderung der ADAMTS13-Aktivität, für deren Entstehung über 100 Mutationen beschrieben sind. Ursächlich ist eine verminderte Sekretion und/oder eine Aktivitätsverminderung von ADAMTS13. Trigger für einen akuten Schub sind Schwangerschaft, verstärkter Alkoholkonsum, Medikamente (wie z. B. Medikamente gegen Malaria, Gemcitabin, Quetiapin, Cyclosporin, Tacrolimus und andere) und Infektionen. Eine Manifestation im frühen Kindesalter ist mit 50–60 % der Fälle häufig. Einige Patienten werden aber erst in der 3. bis 4. Lebensdekade oder auch später symptomatisch, trotz niedriger ADAMTS13-Spiegel über Jahre [5]. Der älteste beschriebene Patient einer Erstmanifestation einer cTTP war 79 Jahre alt [6].

*I*n dem von uns vorgestellten Fall sprachen alle klinischen Befunde und auch der PLASMIC-Score für eine TTP. Es waren (auch in wiederholten Tests) keine Antikörper gegen ADAMTS13 nachweisbar (0,02 IU/ml), womit eine cTTP zumindest in Erwägung zu ziehen war. Neben Autoantikörpern der IgG-Klasse werden allerdings auch Antikörper der IgA-Klasse beschrieben, die der standardisierten Methode der Antikörperbestimmung entgehen könnten. In der Sequenzierung des *ADAMTS13*-Gens der Patientin wurde jedoch keine genetische Ursache gefunden [7]. Da die Indikation zum Plasmaaustausch unabhängig von der Kausalität der TTP besteht, wurde diese noch vor Bestimmung der ADAMTS13-Aktivität eingeleitet.

## Therapie

Ziel der Behandlung in der Akutphase ist es, die Mikrothrombenbildung in der Gefäßstrombahn, die für die Okklusion der Gefäße verantwortlich ist, zu unterbinden. Eine Behandlung sollte daher in den ersten 4–8 h nach der Verdachtsdiagnose einer TTP eingeleitet werden. Der Plasmaaustausch gilt bislang als wichtigste Säule der Therapie. Er trägt zu einer Reduktion der hohen Letalität der TTP von 72–94 % auf 10–20 % bei. Ziel des Plasmaaustauschs ist die Zufuhr von exogener ADAMTS13-Aktivität über Frischplasmen, die sich so vor allem in Abwesenheit von Antikörpern schnell normalisieren lässt [8]. Liegen Antikörper vor, führt der Plasmaaustausch zudem zu einer Elimination von ADAMTS13-neutralisierenden Antikörpern. In der Folge kommt es zu einem Wiederanstieg der Thrombozytenzahl. Die alleinige Plasmainfusion ist bei der aTTP gegenüber dem Plasmaaustausch im Hinblick auf die 6‑Monats-Sterblichkeit unterlegen [9]. Die Plasmainfusion wird aber vor allem bei der cTTP im akuten Schub und als Strategie zur langfristigen Erhöhung der ADAMTS13-Aktivität eingesetzt. Bei fehlender sofortiger Möglichkeit eines Plasmaaustauschs könnte eine Hochdosisplasmainfusion mit 25–30 ml/kg Körpergewicht eine Überbrückungsoption darstellen [10]. Mittlerweile wird rekombinant hergestelltes ADAMTS13 zur Behandlung der cTTP in Studien untersucht [11].

Die Hemmung der Mikroangiopathie gelingt durch Hemmung der Bindung von Thrombozyten an die pathognomonischen ultragroßen von-Willebrand-Faktor-Multimere. Diese wird durch den Nanoantikörper Caplacizumab erreicht, der die Adhäsion von Thrombozyten, konsekutiv die Thrombusformation und damit den Endorganschaden reduziert [12, 13]. Nach Empfehlungen der *International Society on Thrombosis and Haemostasis* (ISTH) sollte die Gabe von Caplacizumab in Erwägung gezogen werden, wenn die Wahrscheinlichkeit für das Vorliegen der Erkrankung als hoch eingeschätzt wird (d. h.: PLASMIC Score > 6), also noch bevor die Erkrankung durch den Nachweis der erniedrigten ADAMTS13-Aktivität zweifelsfrei bestätigt wird. Konsekutiv kommt es unter der Therapie innerhalb weniger Tage – teilweise innerhalb von 24–48 h – zu einer Normalisierung der Thrombozytenzahl, was als gutes Ansprechen auf die Therapie interpretiert werden kann.

Weiteres Standbein der Behandlung einer aTTP ist die Kontrolle der antikörperproduzierenden B‑Zellen. Dazu werden Steroide (z. B. initial 100 mg Prednisolon pro Tag) eingesetzt, mit denen jedoch nicht in allen Fällen eine nachhaltige Kontrolle der antikörperproduzierenden B‑Zellen erreicht wird [3, 14]. Daher hat sich in den letzten Jahren in den allermeisten Zentren die zusätzliche Gabe von Rituximab etabliert, bei dem es sich um einen humanisierten monoklonalen Antikörper gegen das CD20-Antigen auf B‑Zellen handelt. Er wird bei einer Reihe von Autoimmunerkrankungen eingesetzt und ist bei der aTTP wirksam zum Erreichen der Remission und zur Verhinderung von Rezidiven [15, 16]. Allerdings erfolgt der Einsatz von Rituximab außerhalb der arzneimittelrechtlichen Zulassung („off-lable use“).

Patienten mit einer aTTP werden regelhaft mit diesen 3 Behandlungsansätzen therapiert, während bei der cTTP eine Therapie mit Steroiden/Rituximab nicht sinnvoll ist [17].

**Figure 2:**
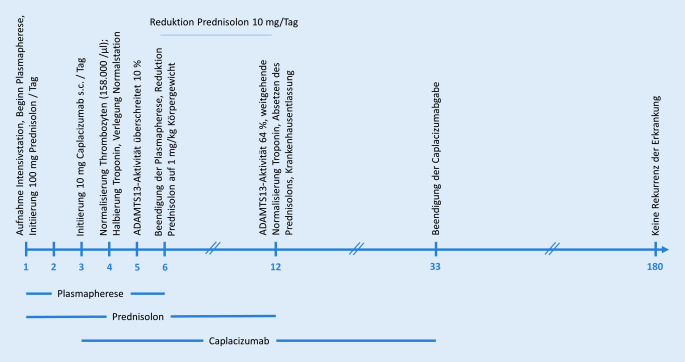

Die von uns vorgestellte Patientin mit einer aTTP wurde parallel zum am Aufnahmetag (Tag 1) eingeleiteten Plasmaaustausch mit 100 mg Prednisolon und ab dem 3. Tag mit 10 mg Caplacizumab s.c. therapiert. Auf eine Rituximabgabe wurde aufgrund des schnellen Ansprechens der Therapie verzichtet (Abb. 2).

### Caplacizumab und Plasmaaustausch

Der Plasmaaustausch dient der exogenen Zufuhr von ADAMTS13 und reduziert zirkulierende ADAMTS13-Antikörper. Caplacizumab verhindert die Bindung von Thrombozyten an die von-Willebrand-Faktor-Multimere, die Thrombusformation und damit den Endorganschaden. Damit sollten beide Therapiestrategien synergistisch wirken und parallel zum Einsatz kommen. Dass Caplacizumab im vorliegenden Fall erst am Tag 3 zum Einsatz kam, lag an der fehlenden unmittelbaren Verfügbarkeit zu diesem Zeitpunkt. Mittlerweile zeigen „Real-world-Daten“ aus Deutschland, Frankreich und England die deutliche Überlegenheit einer caplacizumabbasierten Strategie im Vergleich zur konservativen Strategie im historischen Patientenkollektiv [18–20].

Ermutigt durch diese positiven Ergebnisse hat sich eine Diskussion um die Notwendigkeit und den Stellenwert des Plasmaaustauschs bei der Behandlung der TTP entwickelt. Könnte man unter Einsatz von Caplacizumab zukünftig auf den Plasmaaustausch gänzlich verzichten? In einer Fallserie von 6 Patienten mit aTTP aus Deutschland und Österreich wurde die Notwendigkeit des Plasmaaustauschs zusätzlich zur Caplacizumabgabe untersucht [21]. Die unmittelbare Gabe von Caplacizumab nach Diagnosestellung führte zu einem schnellen und deutlichen Anstieg der Thrombozytenzahl (Verdopplung: 17 h, Normalisierung: 84 h) bereits mit der ersten Dosis. Parallel kam es zu einem Abfall der Laktatdehydrogenase als Ausdruck einer sich auflösenden Mikroangiopathie. Die Autoren schlussfolgern in Analogie zu einer vorhergehenden Untersuchung [22], dass die alleinige Gabe von Caplacizumab eine Alternative zum Plasmaaustausch sein könnte. Allerdings erfordert eine solche Entscheidung große Kenntnisse und Erfahrung in der Diagnose, Differenzialdiagnose und Therapie der TTP sowie die rasche Verfügbarkeit der ADAMTS13-Aktivitätsbestimmung. Bislang wurde Caplacizumab nur zusätzlich zum Plasmaaustausch und zur Immunsuppression in den Zulassungsstudien TITAN und HERCULES untersucht und entsprechend zugelassen.

## Differenzialdiagnostik

### aTTP vs. cTTP

Durch den fehlenden Nachweis von ADAMTS13-Antikörpern auf der einen Seite und den fehlenden Nachweis einer Mutation im *ADAMTS13*-Gen bleibt in unserem vorgestellten Fall die letztliche Differenzierung zwischen einer aTTP (typisch: Antikörper) und der cTTP (typisch: Fehlen von Antikörpern; ideal: Mutationsnachweis) schwierig. Der späte Erkrankungsbeginn mit 49 Jahren und der fehlende genetische Nachweis einer cTTP ließ uns trotz des fehlenden Nachweises von Antikörpern von einer aTTP ausgehen. Therapeutisch unterscheiden sich aTTP und cTTP im Hinblick auf die Notwendigkeit eines sofortigen Plasmaaustauschs (ggf. bei cTTP nur Plasmainfusion) nicht, während die Gabe von Prednisolon und Rituximab nur bei der aTTP sinnvoll ist. Wir entschieden uns für die Gabe von Prednisolon und aufgrund des schnellen Anstiegs der ADAMTS13-Aktivität mit dem Plasmaaustausch gegen den Einsatz von Rituximab.

### Atypisches hämolytisch-urämisches Syndrom (aHUS)

Beim aHUS basiert die TMA auf dem Vorliegen einer genetisch bedingten, chronisch-unkontrollierten Komplementaktivierung. Die Organdysfunktion manifestiert sich beim aHUS häufig über ein ausgeprägtes Nierenversagen. Während der Plasmaaustausch bei der TTP Mittel der Wahl ist, führt sie beim aHUS meist nicht zum gewünschten Therapieerfolg. Eine Komplementinhibition (Eculizumab/Ravulizumab) ist Mittel der Wahl. Die Differenzialdiagnose im Notfall basiert auf Ausschluss einer TTP und STEC-HUS (siehe im Folgenden; Ausschlussdiagnose). Die Durchführung einer Nierenbiopsie mit Nachweis der typischen histologischen Veränderungen einer TMA kann hilfreich sein. Wegen der häufig bestehenden Thrombozytopenie und dem damit verbundenen Blutungsrisiko bietet sich eine transjuguläre Nierenbiopsie in dieser Situation an. Das Ausmaß der Thrombozytopenie kann als Unterscheidungsmerkmal zwischen HUS und TTP genutzt werden. Während bei der TTP die Thrombozytopenie sehr ausgeprägt und häufig < 30.000/µl liegt, ist diese bei HUS nur mittelgradig ausgeprägt, dafür besteht in 100 % aller Fälle eine Nierenbeteiligung.

### Typisches hämolytisch-urämisches Syndrom (STEC-HUS)

Der Diagnose eines STEC-HUS liegt eine Infektion mit Shiga-Toxin-produzierenden Bakterien, zumeist *Escherichia coli*, zugrunde. Definitionsgemäß besteht immer ein akutes Nierenversagen. Kinder sind mit einer Inzidenz von 2 pro 100.000 Einwohner pro Jahr häufiger betroffen. Nach einem Prodromalstadium mit zumeist blutigen Diarrhöen und starken abdominellen Beschwerden manifestiert sich das STEC-HUS als komplementvermittelte systemische TMA. Neurologische Symptome unterschiedlichen Ausmaßes manifestieren sich in etwa 20–25 % aller Fälle und gehen regelhaft mit einer ungünstigeren Prognose einher. Der Nachweis des Shiga-Toxins muss anhand einer Stuhlprobe erreicht werden. Therapeutisch ist die hämodynamische Stabilisierung, die Elektrolytkontrolle und ggf. eine Nierenersatztherapie zielführend. Die Prognose ist allgemein gut, es können jedoch residuale Störung der Nierenfunktion, arterielle Hypertonie oder Proteinurie verbleiben.

Von der TTP unterscheidet sich das STEC-HUS durch eine normale ADAMTS13-Aktivität und den Nachweis von Shiga-Toxin in der Stuhlprobe. Beide Befunde waren im vorliegenden Fall nicht gegeben.

### Andere, sekundäre Formen der TMA

Ätiologisch ist die TMA ein sehr heterogenes Krankheitsbild und ist gemeinsame Endstrecke ganz unterschiedlicher Erkrankungen (Tab. 3). Allen gemeinsam ist die Endothelschädigung der Arteriolen und Kapillaren mit verstärkter Inflammation und Prokoagulation und der typischen Laborkonstellation einer TMA. Obwohl die klinischen, histologischen und Laborborbefunde ähnlich sind wie bei der TTP und HUS, lassen sich bei den sog. sekundären Formen der TMA die typischen Auslöser, wie ADAMTS13-Mangel, Komplementdefekte oder STEC, nicht nachweisen. Als Trigger der sekundären TMA zählen Infektionen, Autoimmunerkrankungen, Transplantation, Medikamente und seltene Stoffwechselerkrankungen wie die schwere Cobalamindefizienz.

**Table 3:** 

Erkrankung	Laborbefund/Pathogenese	Häufigkeit	Klinische Besonderheiten
aTTP	ADAMTS13-Aktivität < 5–10 %, meist Antikörper nachweisbar	Inzidenz 3,1/1 Mio. Einwohner und Jahr (USA)	Neurologie, Petechien, kardiovaskuläre Beteiligung, Nierenbeteiligung
cTTP	ADAMTS13-Defizit, autosomal-rezessiv	Sehr selten, ca. 65 Familien in Deutschland beschrieben	Erstdiagnose zu 50 % im Kindesalter, Schwangerschaft als Trigger, Wundheilungsstörungen
Typisches HUS	Shiga-Toxin-assoziiert (STEC-HUS) Streptokokkenassoziiert	Selten, häufiger Kinder	Nierenversagen, blutige Diarrhöen bzw. Sepsis/Meningitis
Atypisches HUS	Chronische, genetisch bedingte Komplementaktivierung	0,11/1 Mio. Einwohner und Jahr (Erwachsene, Europa)	Nierenbeteiligung häufig im Vordergrund, Rezidive
HELLP (Prä‑)Eklampsie	Transaminasenerhöhung	HELLP: 0,5–0,9 % aller Schwangerschaften	Epileptische Anfälle, Hypertonus, Beendigung durch Entbindung
		TMA: 5–10 % aller Patienten mit schwerer Eklampsie	
Cobalamin-C-Defekte	Homozygote MMACHC-Mutation	Extrem selten, zumindest Kinder < 1 Jahr	Vitamin B_12_-, Folsäuresubstitution
Gerinnungsabhängige TMA	Thrombomodulin‑, DGKE-, Plasminogenmutationen	Extrem selten, zumindest Kinder < 1 Jahr	–
Sekundäre TMA	Autoimmunerkrankungen, Malignome, HIV, Medikamente, maligne Hypertonie	Selten	Unterschiedliche Präsentation, Behandlung der Grunderkrankung im Vordergrund

*aTTP* erworbene thrombotisch-thrombozytopenische Purpura, *cTTP* kongenitale TTP, *HUS* hämolytisch-urämisches Syndrom, *STEC* „Shiga toxin-producing *E. coli*“, *ADAMTS13* „a disintegrin and metalloproteinase with a thrombospondin type 1 motif, member 13“, *HELLP* „hemolysis, elevated liver enzymes, low platelet count“, *MMACHC* „methylmalonic aciduria and homocystinuria type C protein“, *DGKE* Diacylglycerolkinase ε, *HIV* humanes Immundefizienzvirus

## Fazit und Empfehlung für die Praxis

Das Vorliegen einer TTP als Kombination aus Thrombozytopenie, LDH-Erhöhung und Anämie in Kombination mit einer Organbeteiligung ist eine seltene, aber lebensbedrohliche Erkrankung, die unbehandelt mit einer extrem hohen Letalität in der Akutphase einhergeht. Ein erhöhtes Bewusstsein für die Erkrankung und die zielgerichtete weiterführende Diagnostik mit Bestimmung des PLASMIC-Scores bzw. der ADAMTS13-Aktivität führen ggf. direkt zur TTP, deren verzögerte Diagnostik regelhaft zu Sekundärkomplikationen führt, die wiederum eine deutlich erhöhte Morbidität und Mortalität (Letalität) zur Folge haben. Die differenzialdiagnostische Abgrenzung zu anderen Ursachen der TMA ist stets erforderlich. Mit Caplacizumab, Plasmaaustausch, Steroiden sowie Rituximab stehen zur differenzierten Behandlung der TTP heute gute Behandlungsstrategien zur Verfügung, die zu einer deutlichen Verbesserung der Prognose beitragen, vorausgesetzt die klinischen Befunde sowie die Laborkonstellation werden in der akuten Erkrankungsphase korrekt interpretiert und die Therapie ohne Verzögerung eingeleitet (Abb. 3).

**Figure 3:**